# Characterization of the Tumor Immune Microenvironment in Lung Squamous Cell Carcinoma Using Imaging Mass Cytometry

**DOI:** 10.3389/fonc.2021.620989

**Published:** 2021-04-01

**Authors:** Ran Li, Ying Lin, Yu Wang, Shaoyuan Wang, Yang Yang, Xinlin Mu, Yusheng Chen, Zhancheng Gao

**Affiliations:** ^1^ Department of Respiratory and Critical Care Medicine, Peking University People’s Hospital, Beijing, China; ^2^ The Shengli Clinical Medical College, Fujian Medical University, Fuzhou, China; ^3^ Life Science Institute, Jinzhou Medical University, Jinzhou, China; ^4^ Beijing Gencode Diagnostics Laboratory, Beijing, China; ^5^ Department of Respiratory and Critical Care Medicine, Fujian Provincial Hospital, Fuzhou, China

**Keywords:** lung squamous cell carcinoma, tumor immune microenvironment, imaging mass cytometry, CD3^−^CD4^+^ cells, tumor–immune interaction

## Abstract

**Background:**

Lung squamous cell carcinoma (LUSC) is a major subtype of non-small cell lung cancer. The tumor immune microenvironment (TIME) affects the anti-tumor immune response and the patient’s prognosis, although the TIME in LUSC patients is incompletely understood.

**Methods:**

We retrospectively collected surgical specimens from patients with previously untreated primary LUSC. Histopathological examination was used to identify tumor regions and adjacent regions, and imaging mass cytometry was used to characterize the immune cells in those regions. The results were compared between regions and between patients.

**Results:**

We identified heterogeneity in the TIME on comparing different patients with LUSC, although the tumor region and adjacent region both exhibited an immune response to the tumor. The TIME typically included a large number of infiltrating and activated T-cells, especially CD8^+^ T-cells, which closely interacted with the tumor cells in the tumor region. There was limited infiltration of B-cells, NK cells, and NKT cells, while the major immune suppressor cells were CD33^+^ myeloid-derived cells. We also identified a novel population of CD3^−^CD4^+^ cells with high expression of Foxp3 and TNFα, which might modulate the tumor microenvironment and play a proinflammatory role in the TIME.

**Conclusions:**

The TIME of LUSC appears to be immunogenic and heterogenous, with predominant infiltration of activated CD8^+^ T-cells. The interactions between the tumor cells and T-cells facilitate the anti-tumor activity. A novel subpopulation of CD3^−^CD4^+^ cells with high TNFα and Foxp3 expression may modulate the tumor microenvironment and play a proinflammatory role.

## Introduction

Lung cancer is the leading cause of cancer-related death worldwide. Lung squamous cell carcinoma (LUSC) accounts for approximately 30% of non-small cell lung cancer (NSCLC) cases and is usually associated with cigarette smoking (1). Unfortunately, chemotherapy and radiotherapy do not substantially affect the prognosis of patients with LUSC (2). Furthermore, only a limited number of patients with advanced LUSC can benefit from targeted therapy. Immunotherapy using immune checkpoint inhibitors (ICIs) has recently improved the survival of patients with advanced LUSC, and changes in the tumor immune microenvironment (TIME) affect the efficacy of and acquired resistance to ICIs therapy (3).

The TIME is a heterogeneous entity that involves various immune cells and a broad spectrum of chemokines and cytokines. Tumor-infiltrating immune cells play a vital role in the tumor–immune interactions and are useful for predicting patient survival. Regardless of pathological classification, a favorable prognosis is expected in lung cancer patients whose TIME includes significant populations of CD8^+^ T-cells, the M1 subtype of macrophages, tertiary lymphoid structures, B-cells, dendritic cells, and mast cells. In contrast, a poor prognosis is associated with significant populations of regulatory T-cells (Treg cells), the M2 subtype of macrophages, and polymorphonuclear myeloid-derived suppressor cells (MDSCs). Moreover, conflicting results have been reported regarding the prognostic implications of the total numbers of macrophages and Th1 cells in the TIME (4). There is accumulating evidence that tumor-infiltrating lymphocytes (TILs) are a valid biomarker for ICI response and a predictor of survival among patients with NSCLC (1, 5). Furthermore, gene signature data have revealed that higher fractions of resting mast cells and CD4^+^ T-helper cells are associated with longer overall survival, while higher fractions of M2 macrophages and activated dendritic cells are associated with shorter survival in NSCLC patients (6).

Despite these findings regarding NSCLC, the TIME is incompletely understood in LUSC patients. Early lung squamous carcinogenesis involves a predominant increase in neutrophils and the macrophage subtypes in the LUSC tissues (vs. normal tissues) with a shift from resting to activated CD4^+^ T-cells (7). There is also evidence that more CD8^+^ TILs are detected in the tumor nests of LUSC patients, relative to in other types of NSCLC (8). At the molecular level, immune-related gene expression is also correlated with the prognosis of LUSC patients, which includes the gene profile of lymphocytes, NK cells, and macrophages (9–13). However, there is conflicting evidence regarding whether the characteristics of the TIME are associated with the patient’s prognosis (14, 15).

Mass cytometry (cytometry by time-of-flight) is a novel immunophenotyping technique that is similar to flow cytometry, although the antibodies are tagged with heavy metal molecules, which allows for simultaneous detection of multiple cellular markers. In lung adenocarcinoma, mass cytometry has revealed that the TIME has significantly altered T-cell and NK cell components, and that tumor-infiltrating myeloid cells can compromise the anti-tumor function of T-cells (16). Imaging mass cytometry (IMC) can provide additional information regarding cellular localization within tissues and can help clarify the spatial interactions between different cell types (17). In addition, IMC has been used to define the metabolite distribution in a lung adenocarcinoma model (18) and to determine that cancer-associated fibroblasts interact with monocytic myeloid cells to induce immune suppression in the TIME of patients with LUSC (19). Therefore, we used IMC to characterize the TIME’s immune atlas in specimens from patients with primary LUSC, which may help clarify the TIME landscape and explore the cancer–immune interactions. The results also identified a novel population of CD3^−^CD4^+^ cells that might modulate the tumor microenvironment.

## Methods

### Patients and Tissues

This retrospective study evaluated surgical specimens from 12 patients with primary LUSC who underwent surgical resection at the Fujian Provincial Hospital (Fujian, China). The patients had not received chemotherapy, radiotherapy, or immunotherapy before the resection. The patients’ clinical characteristics are summarized in **Table 1**. PD-L1 expression of the tumor tissues was evaluated with tumor proportion scores using the Ventana PD-L1 (SP142) assay (**Table 1**). During the follow-up period, hepatic metastases occurred in case 2, 8 months after resection, who received subsequent chemoradiotherapy; case 4 died from tumor recurrence; and the remaining patients survived without tumor recurrence.

**Table 1:** Clinical characteristics of the patients with primary lung squamous cell carcinoma.

Case	Sex	Age (years)	Smoking history	TNM staging	Tumor location		PD-L1 expression
**1**	Male	81	Yes	pT2bN0M0, IIa		Left lower lobe	40%
**2**	Male	46	Yes	pT3N0M0, IIb		Left lower lobe	30%
**3**	Male	73	Yes	pT2aN0M0, Ib		Right lower lobe	<1%
**4**	Male	60	No	pT2bN0M0, IIa		Right lower lobe	20%
**5**	Male	64	No	pT2bN0M0, IIa		Left lower lobe	10%
**6**	Male	64	No	pT2aN2M0, IIIa		Left lower lobe	20%
**7**	Male	64	Yes	pT2bN0M0, IIa		Right upper lobe	60%
**8**	Male	63	Yes	pT1bN0M0, Ia2		Right lower lobe	40%
**9**	Male	54	No	pT1cN2M0, IIIa		Right upper lobe	90%
**10**	Male	59	Yes	pT2bN0M0, IIa		Right middle and lower lobe	70%
**11**	Male	63	Yes	pT1cN0M0, Ia3		Left lower lobe	70%
**12**	Male	66	Yes	pT2aN2M0, IIa		Right middle and lower lobe	40%

Archived formalin-fixed paraffin-embedded tissues from the surgical specimens were obtained from the database of the Department of Pathology at the Fujian Provincial Hospital. All samples were anonymized; the study was conducted in accordance with the Declaration of Helsinki, and the study protocol was approved by the local ethics committee (K2018-12-035). All samples were histopathologically confirmed to be primary LUSC according to the 2015 World Health Organization classification system. The TNM staging was defined based on the 8th edition of the TNM classification and staging system for lung cancer (20). All samples were tested negative for *EGFR* mutations, *ALK* rearrangements, and *ROS1* rearrangements on immunohistochemical staining.

### Imaging Mass Cytometry

**Figure 1:**
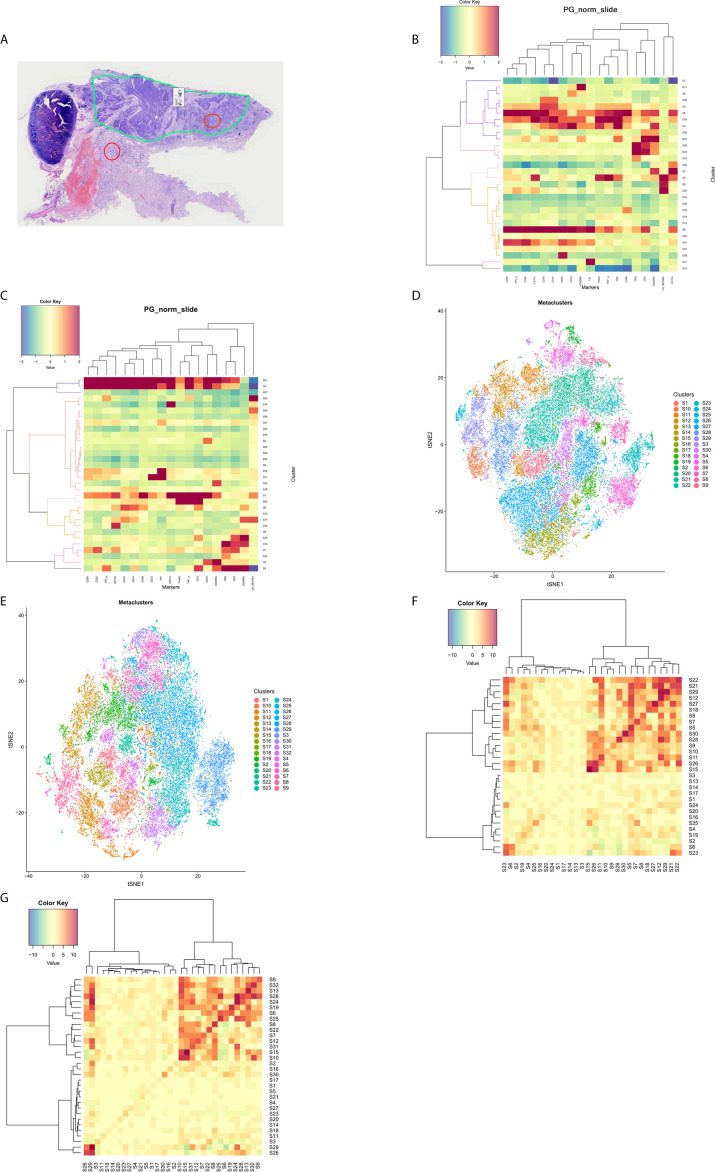
The tumor immune microenvironment in patients with lung squamous cell carcinoma. **(A)** A representative formalin-fixed paraffin-embedded section (4×) was subjected to hematoxylin and eosin staining to identify the tumor region (the loop with green lines) and the adjacent region. Locations within the tumor region (orange circle) and the adjacent region (red circle) were ablated for imaging mass cytometry analysis. PhenoGraph analysis **(B)** and t-distributed stochastic neighbor embedding plots **(D)** were used to divide the cell populations into 30 clusters in the tumor regions according to phenotypic similarity. PhenoGraph analysis **(C)** and t-distributed stochastic neighbor embedding plots **(E)** were used to divide the cell populations into 32 clusters in the adjacent regions according to phenotypic similarity. Neighborhood analysis revealed cell-to-cell interactions between different clusters in the tumor regions **(F)** and the adjacent regions **(G)**, respectively.

The formalin-fixed paraffin-embedded sections were evaluated by a pathologist to determine the general boundary between the tumor region and the adjacent region based on hematoxylin and eosin staining (**Figure 1A**). Two locations (cross-sectional area: 500 μm × 500 μm) within the tumor region and adjacent region were laser-ablated, and data were acquired at 200 Hz for the IMC analysis, which was performed using 21 commercially available antibodies conjugated with metal isotypes (**Table 2**). As the antibodies targeting Ki-67 and CD127 were conjugated to the same metal isotype, samples from five patients (Cases 1–5) were scanned for Ki-67 expression and the remaining samples (Cases 6–12) were scanned for CD127 expression. Images were acquired using a Hyperion Imaging System (Fluidigm) and preprocessing of the raw data was completed using the related acquisition software (Fluidigm). The IMC acquisition stability was monitored based on interspersed acquisition of an isotope-containing polymer (Fluidigm) and image acquisition was considered successful in all cases. Co-expression of various markers and interactions between different cell types were visually observed using pseudo-color images.

**Table 2:** Marker panel for imaging mass cytometry.

Target	Clone	Metal	Vendor	Isotype	Dilution	Description
CD3 CD4 Foxp3 CD8a CD45RA CD45RO CD25 CD19 CD33 CD56 CD68 CD11b/Mac-1 CD11c CD14 CD16 TNFα IFN-***γ*** TdT CK AE1/AE3 CD127 Ki-67	Polyclonal, C-Termina EPR6855 206D D8A8Y MEM-56 UCH-L1 EPR6452 6OMP31 Polyclonal RNL-1 KP1 EPR1344 Polyclonal EPR3653 EPR16784 Polyclonal Polyclonal 7UNA8U6 AE-1/AE-3 EPR2955(2) Ki-67	^170^Er ^156^Gd ^155^Gd ^162^Dy ^143^Nd ^166^Er ^175^Lu ^142^Nd ^145^Nd ^152^Sm ^159^Tb ^149^Sm ^154^Sm ^144^Nd ^146^Nd ^160^Gd ^147^Sm ^141^Pr ^164^Dy ^168^Er ^168^Er	Fluidigm Fluidigm Biolegend Fluidigm Abcam Abcam Abcam Fluidigm Fluidigm Abcam Biolegend Fluidigm Fluidigm Fluidigm Fluidigm Abcam Abcam ebioscience Biolegend Fluidigm Biolegend	Polyclonal IgG IgG1, *κ* IgG IgG2b IgG2a IgG IgG2a IgG IgG1 IgG1, *κ* IgG Polyclonal IgG IgG Polyclonal IgG IgG1, *κ* IgG1, *κ* IgG IgG1, *κ*	50 50 50 50 100 100 50 50 50 50 200 50 50 50 50 50 50 50 400 50 50	T and NKT cells CD4^+^ T cells Treg cells CD8^+^ T cells Naïve T cells Memory T cells IL-2R*α* B cells Myeloid cells NK and NKT cells Macrophages Macrophages Dendritic cells Monocytes NK and monocytes Cytokine Cytokine Immature B, T-cells Tumor biomarker IL-7R*α* Proliferation marker

### Data Analysis

#### Single-Cell Identification

Acquired data were stored in an original MCD file and were visualized using the Fluidigm MCD viewer (version 1.0). Cell population classifications were identified based on the signal values for different markers, as well as the co-localization of the signals with the cells. A minimum signal threshold of three dual counts was set to exclude nonspecific staining or noise. The original MCD file was then converted to TIFF format using imctools (https://github.com/BodenmillerGroup/imctools). The data were segmented into single cells using Cellprofiler (version 3.1.8) (21), which identified single-cell object masks based on the Fluidigm markers for nuclei and the cell surface (*e.g.*, CD3, CD4, or CD8). The masks contained data regarding the cell’s location and boundaries. However, even with very good segmentation, imaging of tissue segments resulted in single-cell data for tissue slices and overlapping cell fragments that did not always capture the nucleus of a cell. Therefore, nuclei-mismatched signals were occasionally assigned to neighboring cells in densely packed areas, which could rarely lead to data from one cell being assigned to a neighboring cell.

#### Data Transformation and Normalization

The marker expressions at the single-cell level were measured using histoCAT software (version 1.75) (22), multiplied by a factor of ×10^7^ to yield values that were >1, and then log-transformed. The single-cell data were censored at the 95th percentile to remove outliers, and z-scored cluster mean values were visualized using heatmaps. The t-distributed stochastic neighbor embedding (t-SNE) and PhenoGraph analyses were performed using data that were normalized using Harmony software (23).

#### Clustering

Single cells were clustered into groups based on their phenotypic similarity using PhenoGraph software (version 2.0) (24). The resulting clusters were aggregated into larger groups based on hierarchical clustering of their mean marker correlations (Euclidean distance and Ward’s linkage). Multiscale bootstrap resampling was used to assess the uncertainty of each subtree (R package pvclust, version 2.0).

#### Barnes-Hut t-SNE

For visualization, the high-dimensional single-cell data were reduced into two dimensions using t-SNE (a nonlinear dimensionality reduction algorithm). We applied the Barnes-Hut implementation of t-SNE to Harmony-normalized data with default parameters.

#### Neighborhood Analysis

Significantly enriched or depleted pairwise neighbor–neighbor interactions between cell types were identified using the CellProfiler MeasureObjectNeighbors module and neighborhood functions (https://github.com/BodenmillerGroup/neighbouRhood), which applies a permutation test-based analysis of spatial single-cell neighborhoods. Neighboring cells were defined as those within four pixels (4 µm). The cell-to-cell distance was determined according to a previously reported algorithm to calculate the probability of co-occurrence (25).

### Comparisons of Different Cell Subtypes

We estimated the numbers and fractions of each cell subtype within the different regions and for each patient. These results were compared using IBM SPSS Statistics software (version 24.0), with Student’s t-test applied for normally distributed data and the Wilcoxon rank-sum test applied for non-normally distributed data. Differences were considered statistically significant at *P*-values of <0.05.

## Results

### Abundant Activated T Cells Were Identified in the TIME and Interacted With Tumor

In the tumor regions, a variety of immune cell components existed in the TIME (**Supplementary Figure 1**). The cell populations were divided into 30 clusters across all samples (S1–S30) using PhenoGraph analysis (**Figure 1B**) and a t-SNE plot was generated to visualize the cluster distributions (**Figure 1D**). Tumor cells with high cytokeratin AE1/AE3 expression were included in clusters S2, S6, and S23. Abundant T-cells had been recruited into the tumor regions, which were mainly CD8^+^ T-cells that had been activated (mostly CD45RO^+^, partially IFN*γ* ^+^) (clusters S24 and S29). Some CD45RA^+^CD45RO^+^CD8^+^ T-cells were observed, which indicated a transition from effector T-cells to memory T-cells. The CD4^+^ T-cells were also predominantly activated (clusters S12 and S28), although CD25^+^Foxp3^+^ Treg cells were dominant in one patient (Case 8). Most patients only had small numbers of infiltrating B-cells, NK cells, and NKT cells, and only one sample exhibited abundant infiltration of B-cells (Case 5). Among the myeloid-derived cells (clusters S3, S4, S5, and S19), a fraction of monocytes/macrophages expressed TNFα, which indicated an activated status. Some CD68^+^ macrophages expressed both CD11b and CD11c, which reflected an M1-like subtype. A large number of CD33^+^ cells were also found to constitute the majority of immunosuppressive cells. Some samples included large numbers of CD19^+^CD33^+^CD56^+^ cells with unknown function. The clusters without expression of immune or tumor markers (“cold clusters”) were considered stromal cells, such as vascular endothelial cells and fibroblasts. The neighborhood analysis (**Figure 1F**) revealed that tumor cells (S23) interacted with CD8^+^ T-cells (S24 and S29) and CD4^+^ T-cells (S12), and that CD8^+^ T-cells (S29) interacted with CD4^+^ T-cells (S12 and S28) to facilitate the co-activation of both subtypes.

In the adjacent regions, we identified 32 clusters that were distinct from those in the tumor regions (**Figures 1C, E** **)**. Tumor cells were scattered in the adjacent regions (clusters S23 and S29) and the most common subpopulations of immune cells in the adjacent regions were also activated CD4^+^ T-cells and CD8^+^ T-cells (clusters S4, S10, and S15). Similar to the tumor regions, neighborhood analysis in the adjacent regions (**Figure 1G**) revealed that CD8^+^ T-cells (S10) interacted with CD4^+^ T-cells (S15) for co-activation, although there was greater cell-to-cell separation between the tumor cells and T-cells, which suggested a weak tumor–immune interaction.

### Heterogeneity Existed in the TIME Between Different Patients

**Figure 2:**
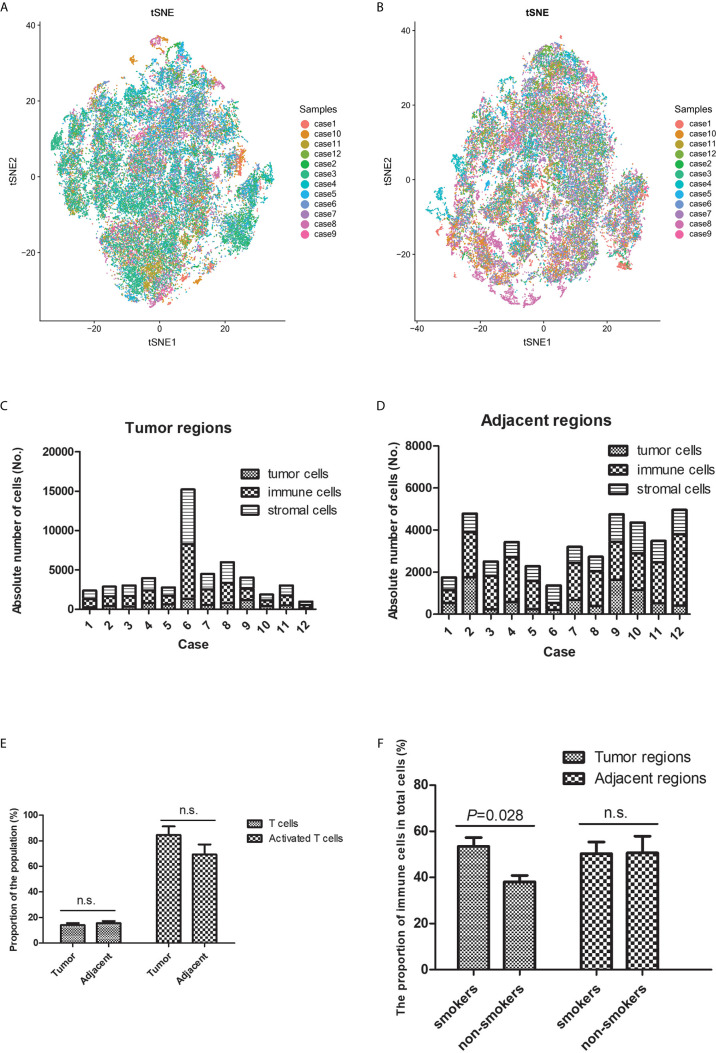
Heterogeneity was observed in the tumor immune microenvironment between patients with lung squamous cell carcinoma. The t-distributed stochastic neighbor embedding plots revealed heterogeneous cell distributions in the tumor **(A)** and adjacent **(B)** regions between different individuals. The immune microenvironment’s cellular components (tumor cells, immune cells, and stromal cells) varied in the tumor **(C)** and adjacent **(D)** regions between individuals. **(E)** There was no significant difference in T-cell infiltration between the tumor and adjacent regions. **(F)** More immune cells tended to infiltrate the tumor regions (rather than the adjacent regions) in smokers, relative to in non-smokers. n.s. means no significance.

The t-SNE plots revealed heterogeneous distributions of the different cell types in the TIME (both tumor and adjacent regions) between different patients (**Figures 2A, B** and **Supplementary Figures 2** and **3**). Tertiary lymphoid structures were identified in seven of the 12 patients (58.3%). Among tumor-infiltrating T-cells, the CD8^+^ cell count was higher than the CD4^+^ cell count in eight of the 12 patients (66.7%), equal CD8^+^ and CD4^+^ cell counts were observed in three patients (25.0%), and a lower CD8^+^ cell count was observed in one patient (8.3%).

The numbers of tumor cells, immune cells, and stromal cells were estimated based on the different phenotypes, which revealed broad heterogeneity in the TIME characteristics between different patients (**Figures 2C, D** **)**. The proportions of T-cells and activated T-cells were not significantly different between the tumor and adjacent regions (**Figure 2E**), which suggested that the immune response was similar near and relatively far away from the tumor nests. A previous study has indicated that the TIME’s composition is influenced by smoking status (15). Thus, we compared the smokers and non-smokers, which revealed more immune cells infiltrating the tumor regions in smokers (**Figure 2F**). However, the subpopulations of T-cells with anti-tumor effects were not significantly different between smokers and non-smokers, which is presumably because more immunosuppressive cells were recruited.

### A Novel Population of CD3^−^CD4^+^ Cells in the TIME

**Figure 3:**
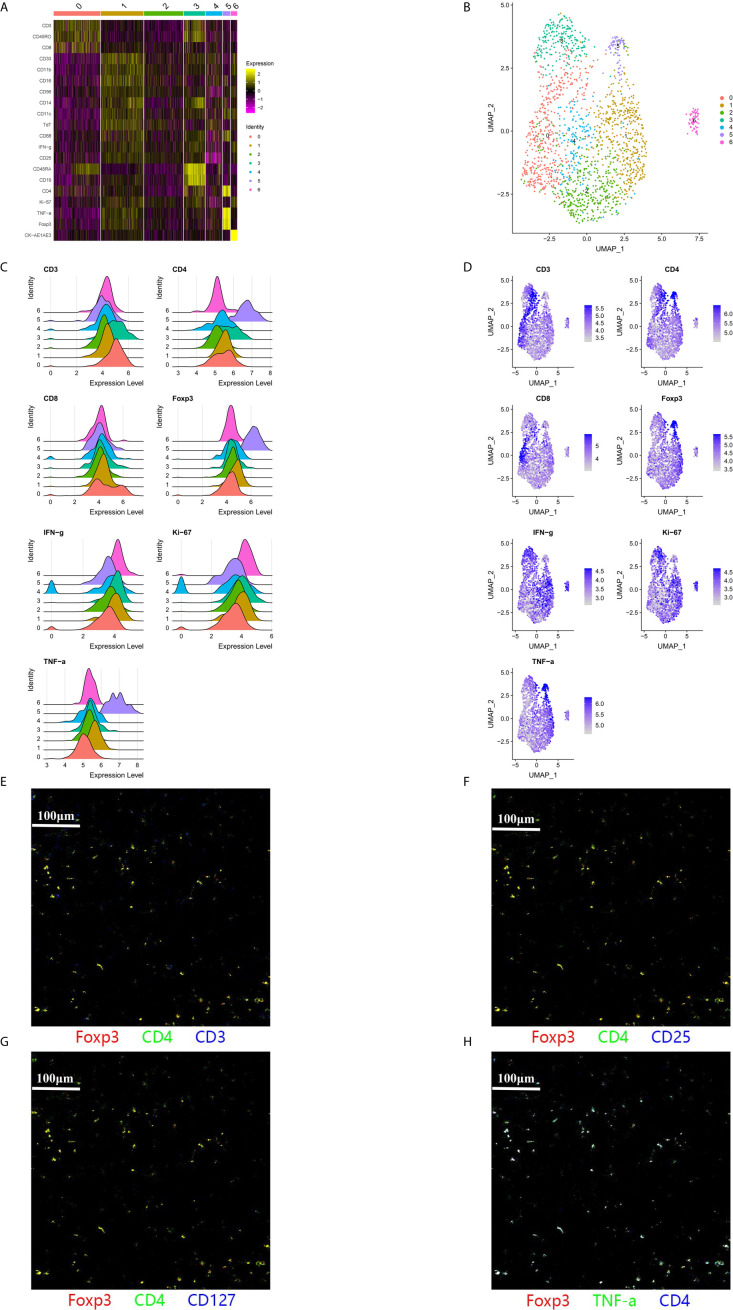
A subpopulation of CD3^−^CD4^+^ cells was identified in the tumor immune microenvironment. **(A–F)** A large population of CD3^−^CD4^+^ cells was identified within the adjacent region of a representative patient (Case 5). Cluster 5 predominantly included CD3^−^CD4^+^ cells with a phenotype of CD3^−^CD4^+^Foxp3^+^TNFα^+^IFN*γ* ^−^. **(G, H)** Pseudo-color images (500 μm × 500 μm) of the adjacent region in another patient (Case 9) also indicated that this CD3^−^CD4^+^ population expressed CD4, Foxp3, and TNFα, without expressing CD3 or CD127.

We identified a novel population of CD3^−^CD4^+^ cells in the tumor region and the adjacent region, which was characterized by a phenotype of CD3^−^CD4^+^Foxp3^+^CD25^−^CD127^−^TNFα^+^IFN*γ* ^−^TdT^+^ (**Figure 3**). Large amounts of CD3^−^CD4^+^ cells were detected in six of the 12 patients (50.0%), although small amounts of CD3^−^CD4^+^ cells were detected in four patients (33.3%), and no CD3^−^CD4^+^ cells were detected in two patients (16.7%). This lymphoid population did not express CD3 and CD25, which is distinct from canonical Treg cells. The high level of TNFα production in this population also indicated that it had a proinflammatory function, rather than being a cluster of anti-inflammatory cells that resemble CD4^+^Foxp3^+^ Treg cells. The CD3^−^CD4^+^ population was also distinct from innate lymphoid cells (ILCs), as most human ILC subtypes have constitutive expression of CD127, with the exception of NK cells (26).

## Discussion

The traditional classification of LUSC is based on the TNM staging system, although patients with the same histological TNM stage can experience significantly different clinical outcomes (4). There is increasing evidence that the TIME is useful for prognostication in various cancer types, although previous studies have frequently used genomic analysis to examine the TIME’s cellular composition. In contrast, IMC can directly evaluate the expression of multiple cellular markers at the single-cell level and facilitate spatial analysis of cell-to-cell interactions.

Our study revealed that the LUSC had an immunogenic TIME, although there was significant heterogeneity in the immune cell composition between different patients. As expected, we observed a large amount of CD45RO^+^CD8^+^ T-cells infiltrating into the tumor region and tertiary lymphoid structures. In this context, CD45RO^+^ T-cells are negatively associated with tumor size, regional metastasis, and TNM stage (27). The lack of B-cells, NK cells, and NKT cells in the TIME suggests that T-cell-mediated cellular immunity played a predominant role in the LUSC patients. In addition, the spatial distribution of different immune cells may influence anti-tumor activity (28), and we observed a strong interaction between tumor cells and T-cells in tumor regions with dense tumor cells, which likely facilitated T-cell activation and anti-tumor activity. Nevertheless, tumor–immune interactions were not detected in the adjacent regions. Although abundant activated T-cells and monocytic cells participate in the anti-tumor immune response, accumulation of CD33^+^ MDSCs creates a suppressor cell population that helps tumor cells escape immune surveillance (29).

Interestingly, we identified a novel population of CD3^−^CD4^+^ cells in the TIME, and we are not aware of any reports that have described this type of cell in the tumor microenvironment. Lymphoid tissue inducer (LTi) cells in the fetal lymph nodes of mice have the CD3^−^CD4^+^ phenotype (30), which belongs to the family of ILCs. Furthermore, CD3^−^CD4^+^ cells support the formation of secondary lymph nodes and also interact with primed and memory T-cells in mice (31). However, human LTi cells lack CD4 expression (32), while human clonal CD3^−^CD4^+^ cells can be found in T-cell lymphomas and lymphoid variants of hypereosinophilic syndrome (33). Normal human subjects were also found to have a population of CD3^−^CD4^+^ cells that express TNF and CD127, and this population is expanded in patients with rheumatoid arthritis (34). Interestingly, while this CD3^−^CD4^+^ population belongs to the T-cell lineage, it is activated by innate signals, such as IL-7, instead of conventional T-cell receptor signaling (34). Populations of CD3^−^CD4^+^ cells can also be found in other autoimmune diseases, including psoriasis, where elevated OX40 expression potentially contributes to the OX40-OX40L interaction and promotes the expansion and survival of effector T-cells (35). The CD3^−^CD4^+^ cells in our study were distinct from the subset with high CD127 expression, although IL-7 stimulation can downregulate CD127 expression in CD3^−^CD4^+^ cells (34). The expression of Foxp3 also suggests that the CD3^−^CD4^+^ cells in our study are a novel subtype without a clearly understood function. Furthermore, because the CD3^−^CD4^+^ cells had high expression of TNFα, we speculate that they function as a proinflammatory cell type.

Our study has some limitations. First, the cell population classifications were estimated using the signal intensities for different markers, which may lead to deviation in instances with modest signal intensities. However, we applied a minimum signal threshold of three dual counts, combined with the observation of signal co-localization in IMC images, to warrant that the identified positive signal is maximally reliable. Second, we did not perform functional testing for the CD3^−^CD4^+^ population with high levels of TNFα production. This CD3^−^CD4^+^ population has not been reported in cancer patients in the literature. As TNFα has dual role of either promoting or inhibiting tumor growth, further studies are needed to characterize the function of this novel cell population in lung cancer. Third, as all patients in our study were in the early stage and most did not experience tumor recurrence so far, we did not investigate the relationship between the immune cell landscape of the TIME and the prognosis of the LUSC patients, which would require a larger sample size and long-term follow-up.

## Conclusions

We used IMC to evaluate the characteristics of the TIME in specimens from patients with primary LUSC and to facilitate the understanding of the spatial relationship among different cell types in the TIME. Activated CD8^+^ T-cells were the predominant cell type infiltrating the tumor bed, which suggested that the anti-tumor immune response mainly involved cell-mediated immunity. The interactions between the tumor cells, CD8^+^ T-cells, and CD4^+^ T-cells also appeared to facilitate the anti-tumor activity. The main immunosuppressive population was CD33^+^ myeloid-derived cells. We identified a novel subpopulation of CD3^−^CD4^+^ cells with high TNFα and Foxp3 expression, which might modulate the tumor microenvironment and played a proinflammatory role.

## Data Availability Statement

The original contributions presented in the study are included in the article/**Supplementary Material**. Further inquiries can be directed to the corresponding authors.

## Ethics Statement

The studies involving human participants were reviewed and approved by the ethics committee of Fujian Provincial Hospital. The ethics committee waived the requirement of written informed consent for participation.

## Author Contributions

RL, YL, YC, and ZG participated in the design of the study. YL collected the clinical information and specimens. RL, YW, SW and YY analyzed and interpreted the data. RL organized the data and drafted the manuscript. YL, XM, YC and ZG contributed to the revision and editing. All authors contributed to the article and approved the submitted version.

## Funding

This study was supported by high-level hospital grants from Fujian Provincial Hospital, Fujian, China (No. 2018GSP008).

## Conflict of Interest

The authors declare that the research was conducted in the absence of any commercial or financial relationships that could be construed as a potential conflict of interest.

## Supplementary Material

Supplementary Figure 1 Representative pseudo-color images of the different cell types identified using imaging mass cytometry. **(A)** CD4^+^ T-cells, **(B)** CD8^+^ T-cells, **(C)** B-cells, **(D)** regulatory T-cells, **(E)** natural killer cells, **(F)** natural killer T-cells, **(G)** monocytes/macrophages, **(H)** M1-like macrophages, **(I)** dendritic cells, **(J)** CD33^+^ myeloid-derived cells, **(K)** CD19^+^CD33^+^CD56^+^ cells, and **(L)** tumor cells.

Supplementary Figure 2 A heatmap of the marker expressions in the tumor regions revealed heterogeneity between individuals. Panels **(A–L)** show Cases 1–12.

Supplementary Figure 3 A heatmap of the marker expressions in the adjacent regions revealed heterogeneity between individuals. Panels **(A–L)** show Cases 1–12.
